# BluePrint molecular subtypes predict response to neoadjuvant pertuzumab in HER2-positive breast cancer

**DOI:** 10.1186/s13058-023-01664-x

**Published:** 2023-06-19

**Authors:** M. C. Liefaard, A. van der Voort, M. S. van Ramshorst, J. Sanders, S. Vonk, H. M. Horlings, S. Siesling, L. de Munck, A. E. van Leeuwen, M. Kleijn, L. Mittempergher, M. M. Kuilman, A. M. Glas, J. Wesseling, E. H. Lips, G. S. Sonke

**Affiliations:** 1 Division of Molecular Pathology, The Netherlands Cancer Institute, Amsterdam, The Netherlands; 2 Department of Medical Oncology, The Netherlands Cancer Institute, Amsterdam, The Netherlands; 3 Department of Pathology, The Netherlands Cancer Institute, Amsterdam, The Netherlands; 4 Core Facility Molecular Pathology and Biobanking, The Netherlands Cancer Institute, Amsterdam, The Netherlands; 5 Department of Pathology, Leiden University Medical Center, Leiden, The Netherlands; 6 Department of Research and Development, Netherlands Comprehensive Cancer Organization (IKNL), Utrecht, The Netherlands; 7 Department of Health Technology and Services Research, Technical Medical Centre, University of Twente, Enschede, The Netherlands; 8 Dutch Breast Cancer Research Group, BOOG Study Center, Amsterdam, The Netherlands; 9 Department of Research and Development, Agendia NV, Amsterdam, The Netherlands

**Keywords:** Molecular subtyping, Predictive biomarkers, Breast cancer, ERBB2, Monoclonal antibodies, Response prediction

## Abstract

**Background:**

The introduction of pertuzumab has greatly improved pathological complete response (pCR) rates in HER2-positive breast cancer, yet effects on long-term survival have been limited and it is uncertain which patients derive most benefit. In this study, we determine the prognostic value of BluePrint subtyping in HER2-positive breast cancer. Additionally, we evaluate its use as a biomarker for predicting response to trastuzumab-containing neoadjuvant chemotherapy with or without pertuzumab.

**Methods:**

From a cohort of patients with stage II-III HER2-positive breast cancer who were treated with neoadjuvant chemotherapy and trastuzumab with or without pertuzumab, 836 patients were selected for microarray gene expression analysis, followed by readout of BluePrint standard (HER2, Basal and Luminal) and dual subtypes (HER2-single, Basal-single, Luminal-single, HER2-Basal, Luminal-HER2, Luminal-HER2-Basal). The associations between subtypes and pathological complete response (pCR), overall survival (OS) and breast cancer-specific survival (BCSS) were assessed, and pertuzumab benefit was evaluated within the BluePrint subgroups.

**Results:**

BluePrint results were available for 719 patients. In patients with HER2-type tumors, the pCR rate was 71.9% in patients who received pertuzumab versus 43.5% in patients who did not (adjusted Odds Ratio 3.43, 95% CI 2.36–4.96). Additionally, a significantly decreased hazard was observed for both OS (adjusted hazard ratio [aHR] 0.45, 95% CI 0.25–0.80) and BCSS (aHR 0.46, 95% CI 0.24–0.86) with pertuzumab treatment. Findings were similar in the HER2-single subgroup. No significant benefit of pertuzumab was seen in other subtypes.

**Conclusions:**

In patients with HER2-type or HER2-single-type tumors, pertuzumab significantly improved the pCR rate and decreased the risk of breast cancer mortality, which was not observed in other subtypes. BluePrint subtyping may be valuable in future studies to identify patients that are likely to be highly sensitive to HER2-targeting agents.

**Supplementary Information:**

The online version contains supplementary material available at 10.1186/s13058-023-01664-x.

## Background

The addition of pertuzumab, a monoclonal antibody that inhibits HER2-HER3 dimerization and activates antibody-dependent cellular cytotoxicity (ADCC), to neoadjuvant treatment with chemotherapy and trastuzumab has significantly improved pathological complete response (pCR) rates in patients with HER2-positive breast cancer [1–3]. However, effects on long-term survival outcomes have been modest, and pertuzumab treatment is associated with increased toxicity and costs [4–8]. Results of previous studies indicate that a subset of patients is highly responsive to neoadjuvant treatment consisting of dual HER2-blockade with trastuzumab and pertuzumab without chemotherapy [1, 9–11]. In contrast, another subset of patients seems to be non-responsive despite dual HER2-blockade. Biomarkers that differentiate patients with high likelihood of response and excellent prognosis from those with poor outcomes may assist in selecting some patients for chemotherapy-free regimens and others for intensified or novel regimens. Although hormone receptor (HR) status has been established as a predictor of pCR in patients with HER2-positive breast cancer who are treated with neoadjuvant HER2-blockade, it does not accurately differentiate between non-responders and responders, as both patients with HR-negative disease and patients with HR-positive disease benefit from pertuzumab [1–3, 5]. While several other potential biomarkers for predicting response to HER2-blocking agents have been investigated, a reliable marker has not been established [12]. Thus, additional prognostic and predictive biomarkers are required to improve treatment decision making.

It is plausible that tumors which rely heavily on the HER2-pathway for their survival and proliferation, so-called HER2-driven or HER2-addicted tumors, are highly sensitive to HER2-blockade. This hypothesis has been supported by studies that show a strong association between the HER2-enriched intrinsic subtype and pCR in patients receiving neoadjuvant dual HER2-blockade [13]. However, an interaction with pertuzumab treatment has not been investigated, and associations with long-term outcomes have not been described.

Intrinsic tumor subtypes can be determined using BluePrint, an 80-gene molecular subtyping test that classifies breast tumors as Basal-, Luminal- or HER2-type based on gene expression analysis [14]. Recent data shows that, although the majority of tumors exhibit a high signature score for one single subtype, in some tumors equally high gene expression scores are observed for more than one subtype, which indicates that multiple pathways are activated [15]. Analysis of tumors that were assigned a ‘dual subtype’ has shown that their biology differs from tumors with a single dominant subtype, which may have implications for treatment response and prognosis [15]. Indeed, secondary analyses from the APHINITY trial (NCT01358877) suggest that pertuzumab benefit is largely restricted to patients with single-activated HER2-type tumors and less pronounced in patients with other single- or dual-activated subtypes [16]. In this study, we evaluated BluePrint standard and dual subtypes as a biomarker for predicting response to trastuzumab-containing neoadjuvant chemotherapy with or without pertuzumab in a large cohort of patients with HER2-positive breast cancer.

## Methods

### Patient data and materials

Data of all patients with stage II or III HER2-positive breast cancer who were treated in the Netherlands with neoadjuvant chemotherapy plus trastuzumab between January 2013 and January 2016 were obtained from the Netherlands Cancer Registry (NCR). This cohort included 438 patients who participated in the TRAIN-2 trial (NCT01996267, registration date November 27, 2013). At the time, pertuzumab was not routinely available to patients treated outside of the trial, resulting in two cohorts of patients that were treated with either single or dual HER2-blockade. Study design, in- and exclusion criteria and results of the TRAIN-2 trial have been previously published [3, 17, 18]. Data on clinical characteristics and pCR were provided by the NCR. Data on date and cause of death were acquired through linkage with Statistics Netherlands (CBS). Pre-treatment formalin-fixed paraffin-embedded (FFPE) biopsy tissues were collected through the nationwide network and registry of histo- and cytopathology in the Netherlands (PALGA) [19]. Tumor grade was scored by a pathologist (J.S.) on hematoxylin–eosin (HE) tissue slides for patients who had missing data using an online platform [20]. Additionally, tumor cell percentage (TCP) was scored on HE slides for all patients by a pathologist (J.S.). Tumors were considered HR positive in case of estrogen receptor (ER) and/or progesterone receptor (PR) positivity, which was defined as ≥ 10% positive nuclear staining, following the Dutch guideline for diagnosis and treatment of breast cancer [21]. Consistent with the 2014 ASCO/CAP guideline, HER2 positivity was defined as overexpression and/or amplification of HER2 in an invasive component of the core biopsy, as > 10% of invasive tumor cells showing strong complete circumferential membrane staining on immunohistochemistry (score 3 +), and/or HER2 gene amplification defined as ≥ 6 HER2 gene copies per nucleus by in situ hybridization [22].

### Gene expression analysis

The study was designed to perform gene expression analysis on biopsies of 836 patients, based on the expectation that sufficient material would be obtained for 418 patients who were treated with dual HER2-blockade, who would be matched to 418 patients that received single HER2-blockade. Tumor blocks with a TCP of 30% or higher and sufficient tumor material available were considered suitable for gene expression analysis. Since the quantity and quality of biopsy material was insufficient in several patients, the number of 418 patients was not reached. Therefore, all 404 patients who received pertuzumab and met the eligibility criteria for tumor tissue were matched using variable ratio matching to 432 control patients who did not receive pertuzumab to reach the pre-specified total of 836 patients, using the MatchIt package version 4.1.0 in R [23]. Matching factors included anthracycline treatment (yes vs. no), age and HR status. For those patients, 10 × 5 um slides were cut and sent to Agendia for RNA isolation and gene expression profiling through microarray according to previously published methods [14, 24]. Technicians at Agendia were blinded for tumor characteristics, treatment arm and outcome. Based on gene expression results, BluePrint standard (HER2-type, Basal-type and Luminal-type) and dual subtypes (HER2-single-type, Basal-single-type, Luminal-single-type, Luminal-HER2-type, HER2-Basal-type, Luminal-Basal-type, Luminal-HER2-Basal-type) were determined according to previously published algorithms [14, 15, 24, 25].

### Endpoints

The primary endpoint was pCR, defined as absence of all invasive tumor cells in the breast and axilla after neoadjuvant treatment (ypT0/isN0). Secondary endpoints were overall survival (OS) and breast cancer-specific survival (BCSS), which were defined as the time between the date of diagnosis of primary breast cancer and the date of death from any cause or the date of last follow-up, and the time between the date of diagnosis of primary breast cancer until the date of death from breast cancer or last follow-up date, respectively.

### Statistical analyses

Missing data were imputed in the entire dataset for 50 times using the mice package version 3.13.0 in R [26]. Clinical T-stage (cT), clinical N-stage (cN), tumor grade and HER2 immunohistochemistry (IHC) scores were imputed using ordered logistic regression, and estrogen receptor status and progesterone receptor status were imputed using logistic regression. For patients with unknown nodal stage after treatment, a pCR was assumed if they had node-negative disease at diagnosis and a pCR in the breast was observed after neoadjuvant treatment. Associations between clinical variables and pCR were assessed by univariable logistic regression. The association between BluePrint standard and dual subtypes and pCR was assessed through multivariable logistic regression, adjusted for variables that were statistically significant in the univariable analyses, variables that were significantly different between the treatment groups, and variables that were known from the literature to potentially be associated with pCR. Standard and dual-type BluePrint subtypes were analyzed as categorical variables, as well as binary variables where all other subtypes besides HER2 were grouped together (non-HER2-type or non-HER2-single-type). Kaplan–Meier curves were constructed, and log-rank tests were performed. Survival analyses were performed with Cox proportional hazard regression, adjusted for the same set of variables as in the analyses with pCR as outcome. BCSS was evaluated using cause-specific hazard models and subdistribution hazard models. To assess potential interactions between molecular subtype and clinical or treatment variables in relation to outcome, additional multivariable logistic and Cox regression analyses were performed in which an interaction term was included. In addition, subgroup analyses were performed. All tests were two-sided, and p-values of < 0.05 were considered statistically significant. Statistical analyses were performed with R version 4.0.5 [27].

## Results

### Clinical characteristics and genomic results

In total, 836 patients were selected for gene expression analysis, of whom 404 were treated with pertuzumab and 432 were not. Microarray was performed successfully for 719 patients. Tumor grade, node-positive disease and anthracycline treatment were significantly different between the two treatment groups (Table 1). Due to missing information on nodal disease at surgery, pCR was missing in 9 patients that received pertuzumab and 11 patients that did not. For 1 patient in the pertuzumab group and 3 patients in the non-pertuzumab group who presented with node-negative disease at baseline and had a pCR in the breast, a pCR was assumed.

**Table 1:** Study population characteristics

	No pertuzumab	Pertuzumab	Overall	p-value
	n = 362	n = 357	n = 719	
Age (years)				
Mean (sd)	51.0 (12.4)	49.3 (9.83)	50.1 (11.2)	0.09
Median (IQR)	49.0 (43.0–61.0)	49.0 (43.0–56.0)	49.0 (43.0–58.0)	
HR status				
Negative	128 (35.4%)	137 (38.6%)	265 (37.0%)	0.39
Positive	234 (64.6%)	218 (61.4%)	452 (63.0%)	
Missing	N/A	2	2 (0.3%)	
Grade				
1–2	131 (36.6%)	175 (49.0%)	306 (42.8%)	< 0.001
3	227 (63.4%)	182 (51.0%)	409 (57.2%)	
Missing	4		4	
cT				0.75
0–2	250 (69.4%)	252 (70.6%)	502 (70.0%)	
3–4	110 (30.6%)	105 (29.4%)	215 (30.0%)	
Missing	2	0	2	
cN				0.007
Negative	106 (29.4%)	138 (40.0%)	244 (34.2%)	
Positive	254 (70.6%)	216 (60.0%)	470 (65.8%)	
Missing	2	3	5	
HER2 IHC				0.23
1+	1 (0.3%)	1 (0.3%)	2 (0.3%)	
2+	28 (9.2%)	19 (6.3%)	47 (7.7%)	
3+	276 (90.5%)	282 (93.4%)	558 (91.9%)	
Missing	57	55	112	
Anthracyclines				0.001
No	134 (37.0%)	175 (49.0%)	309 (43%)	
Yes	228 (63.0%)	182 (51.0%)	410 (57.0%)	
BluePrint Standard Subtype				0.58
HER2-type	308 (85.1%)	313 (87.7%)	621 (86.4%)	
Basal-type	6 (1.7%)	4 (1.1%)	10 (1.4%)	
Luminal-type	48 (13.3%)	40 (11.2%)	88 (12.2%)	
BluePrint Dual Subtype				0.54
HER2-single-type	279 (77.1%)	278 (77.9%)	557 (77.5%)	
Basal-single-type	4 (1.1%)	2 (0.6%)	6 (0.8%)	
Luminal-single-type	31 (8.6%)	29 (8.1%)	60 (8.3%)	
Luminal-HER2-type	40 (11.0%)	41 (11.5%)	81 (11.3%)	
HER2-Basal-type	7 (1.9%)	4 (1.1%)	11 (1.5%)	
Luminal-Basal-type	1 (0.3%)	0 (0%)	1 (0.1%)	
Luminal-HER2-Basal-type	0 (0%)	3 (0.8%)	3 (0.4%)	
pCR				< 0.001
No	205 (59.2%)	113 (33.8%)	318 (46.8%)	
Yes	132 (38.2%)	212 (63.5%)	344 (50.6%)	
Missing	9 (2.6%)	9 (2.7%)	18 (2.6%)	

*HR status* hormone receptor status, *cT* clinical T-stage, *cN* clinical N-stage, *HER2 IHC* HER2 immunohistochemistry score

**Fig. 1:**
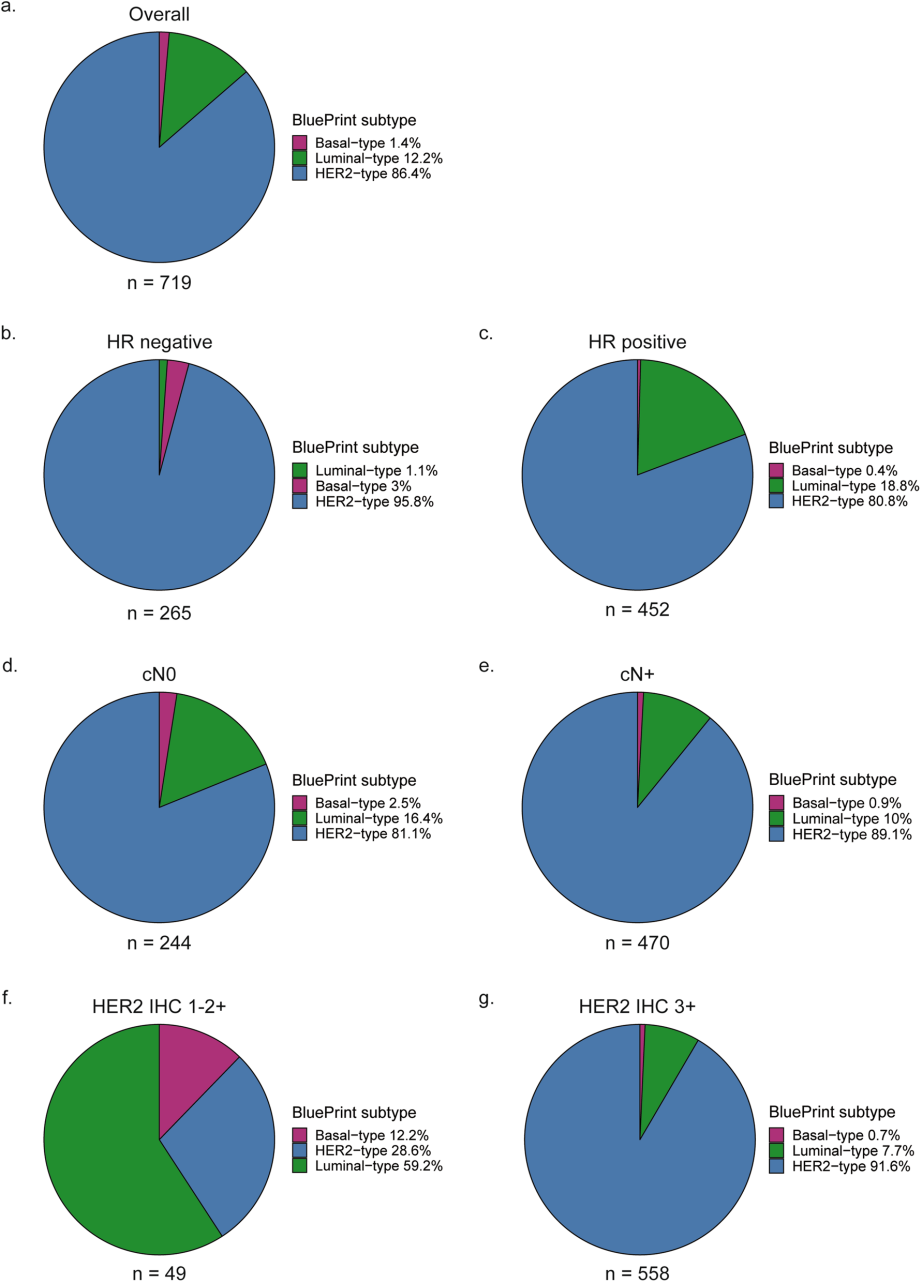
BluePrint standard subtypes and clinical variables **a** Total group, **b** Hormone receptor-negative disease, **c** Hormone receptor-positive disease, **d** Node-negative disease, **e** Node-positive disease, **f** HER2 immunohistochemistry 1–2 + tumors, **g** HER2 immunohistochemistry 3 + tumors. *HR* hormone receptor, *cN0* clinically node-negative, *cN* + clinically node-positive, *HER2 IHC* HER2 immunohistochemistry score

The most prevalent subtype in the total group according to the BluePrint standard readout was HER2-type, followed by Luminal-type and Basal-type (Fig. 1a). Prevalence of BluePrint subtypes differed based on hormone receptor status, clinical N-stage and HER2 IHC score (Fig. 1b-g). In the hormone receptor-positive, node-negative, node-positive and IHC 3 + subgroups, the most common subtype was HER2-type, followed by Luminal-type. In hormone receptor-negative disease, the HER2-type was most prevalent, followed by Basal-type. In IHC 1–2 + tumors, the most common subtype was Luminal-type, followed by HER2-type. Of the 49 IHC 1–2 + tumors, 47 (96%) was HR positive, whereas 337 (60.4%) of the IHC 3 + tumors was HR positive.

**Fig. 2:**
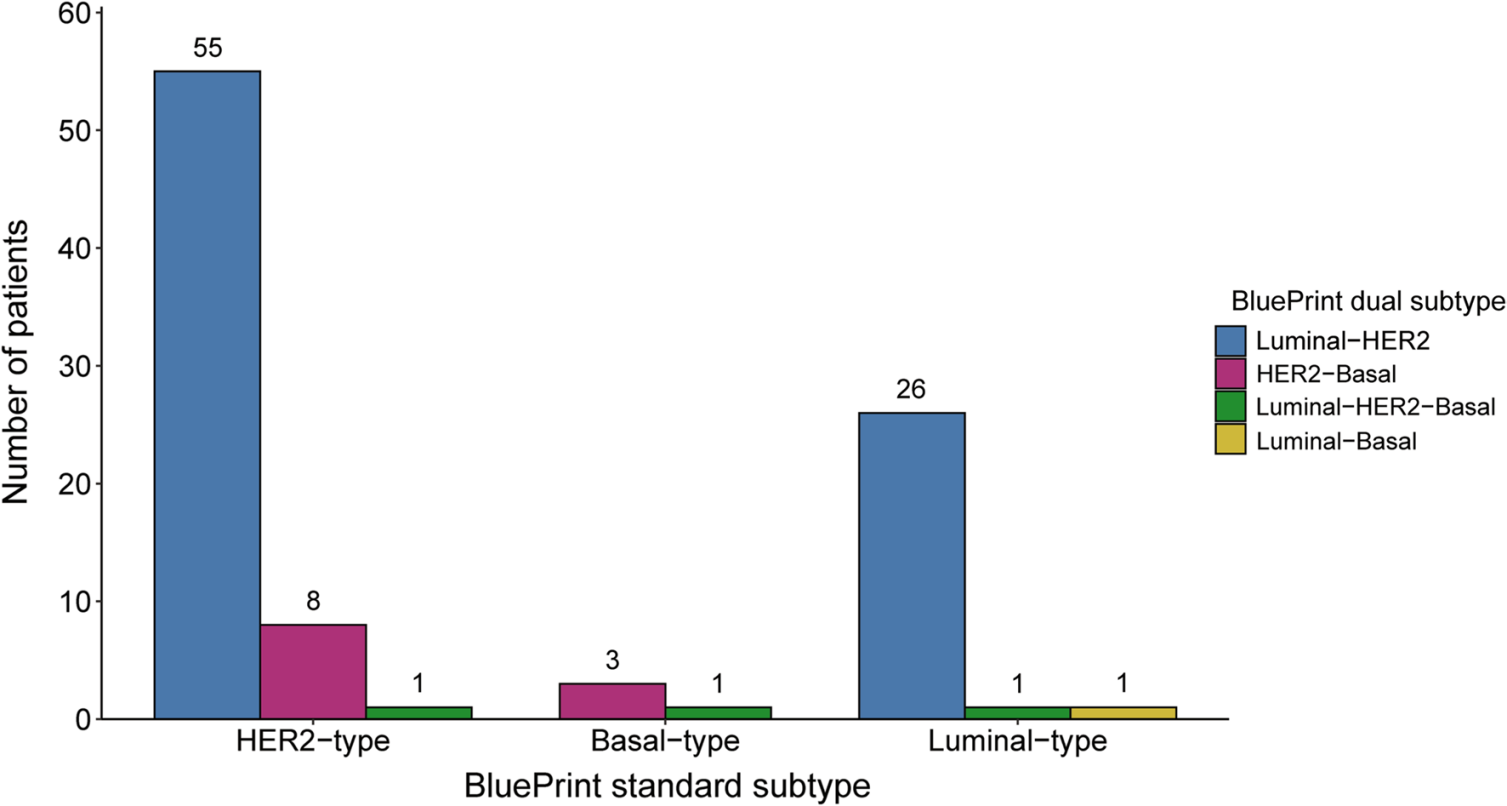
Subtype reclassifications from BluePrint standard subtype to BluePrint dual-subtype readouts

Upon further classification with the dual-subtype readout, 8.9% (n = 55) of HER2-type and 29.5% (n = 26) of Luminal-type tumors were further classified as Luminal-HER2-type (Fig. 2). The most common subtypes besides HER2-single-type were Basal-single-type and HER2-Basal-type in hormone receptor-negative tumors and Luminal-HER2-type and Luminal-single-type in hormone receptor-positive tumors (Additional file 1: Figure S1a-g).

### Association of BluePrint standard readout and treatment with pCR

**Fig. 3:**
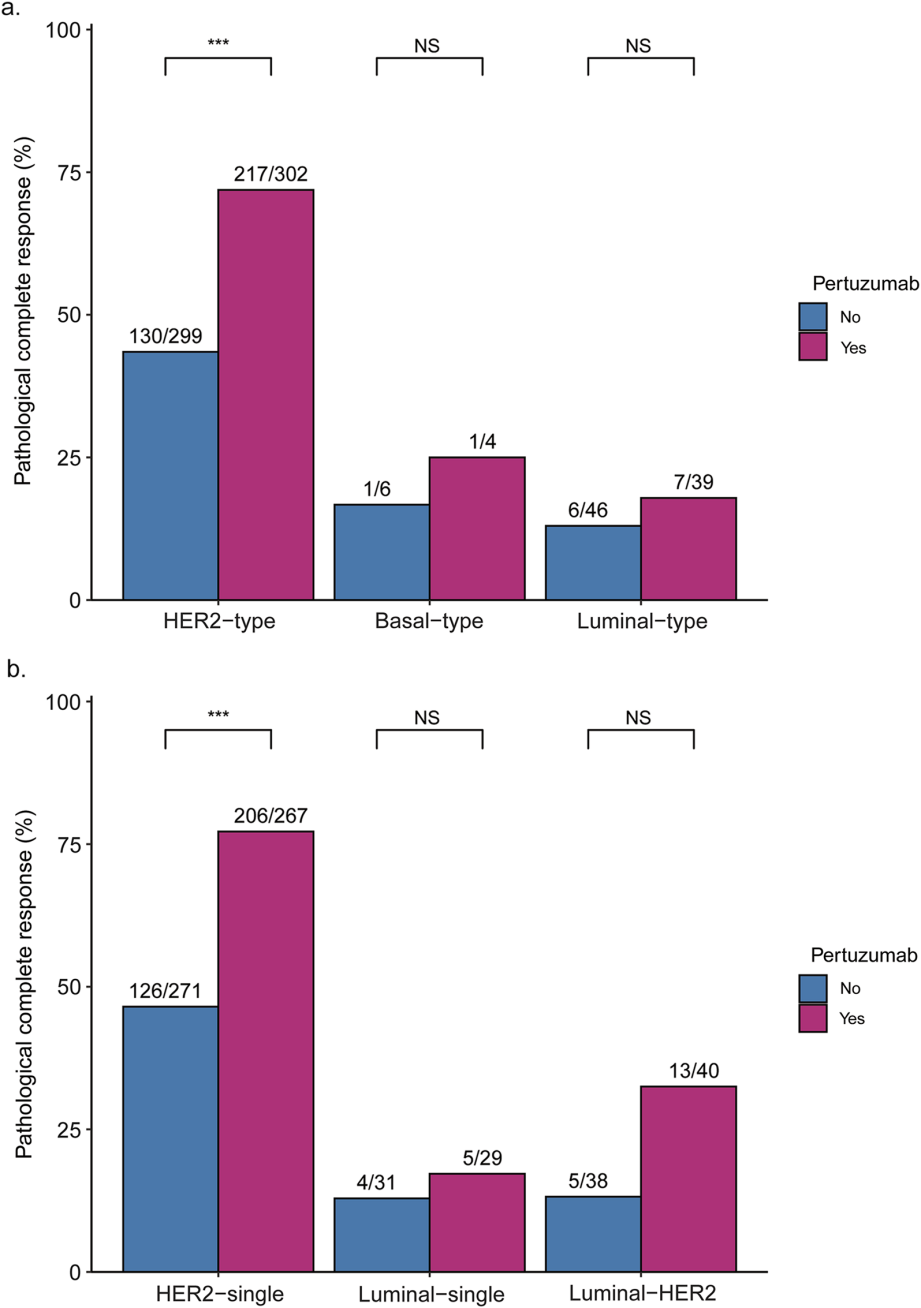
Pathological complete response rates according to molecular subtype. **a** Pathological complete response rates according to pertuzumab treatment for the BluePrint standard subtypes. Patients with Basal- and Luminal-type tumors are grouped together for the non-HER2-type category. **b** Pathological complete response rates according to pertuzumab treatment for the BluePrint dual subtypes. Only subtypes with n > 10 are shown. *** Fisher exact test p-value < 0.001. *NS* not significant

In patients with HER2-type tumors that received pertuzumab, the pCR rate was 71.9% versus 43.5% in patients that did not receive pertuzumab (Fig. 3a). A significant association was seen between BluePrint standard subtype and pCR in a both a univariable as well as a multivariable logistic regression analysis adjusted for age, HR status, tumor grade, HER2 IHC score, clinical T-stage (cT), clinical N-stage (cN), anthracycline treatment and pertuzumab treatment (Additional file 2: Table S1 and S2). When patients with Basal-type tumors and Luminal-type tumors were combined in a non-HER2 subgroup, they had 83% lower odds of reaching pCR compared to patients with HER2-type tumors (Table 2; Additional file 2:Table S2). No significant interaction between BluePrint subtype and either anthracycline or pertuzumab treatment was observed. However, BluePrint standard subtype and HER2 IHC score did show a significant interaction (p = 0.014). In the overall study population, patients with HER2 IHC 3 + tumors, the non-HER2-type was associated with much lower odds of reaching pCR compared to HER2-type tumors (adjusted odds ratio [aOR] 0.09, 95% CI 0.04–0.21, p < 0.001, median n of pooled model = 633). In the group of HER2 IHC 1–2 + tumors, no such association was observed, but the sample size was too small for meaningful results (median n of pooled model = 63). Subgroup analyses based on hormone receptor status showed that the non-HER2 subtype was associated with significantly lower odds of reaching pCR in the HR positive (aOR 0.16, 95% CI 0.08–0.34, p < 0.001, median n of pooled model = 439), but not the HR-negative subgroup (aOR 0.23, 95% CI 0.04–1.34, p = 0.10, median n of pooled model = 256).

**Table 2:** BluePrint standard and dual subtypes in relation to pathological complete response and survival

		Pathological complete response		Overall survival		Breast cancer-specific survival	
		aOR (95% CI)	p	aHR (95% CI)	p	aHR (95% CI)	p
Standard subtype	HER2-type	Ref		Ref		Ref	
	Other	0.17 (0.09–0.32)	< 0.001	1.33 (0.58–3.03)	0.50	1.35 (0.54–3.39)	0.52
Dual subtype	HER2-single-type	Ref		Ref		Ref	
	Other	0.15 (0.09–0.24)	< 0.001	2.04 (1.08–3.84)	0.028	2.38 (1.20–4.70)	0.014

*aOR* adjusted Odds Ratio, *aHR* adjusted Hazard Ratio, *95% CI* 95% Confidence Interval, *p* p-value; *Ref* reference

**Fig. 4:**
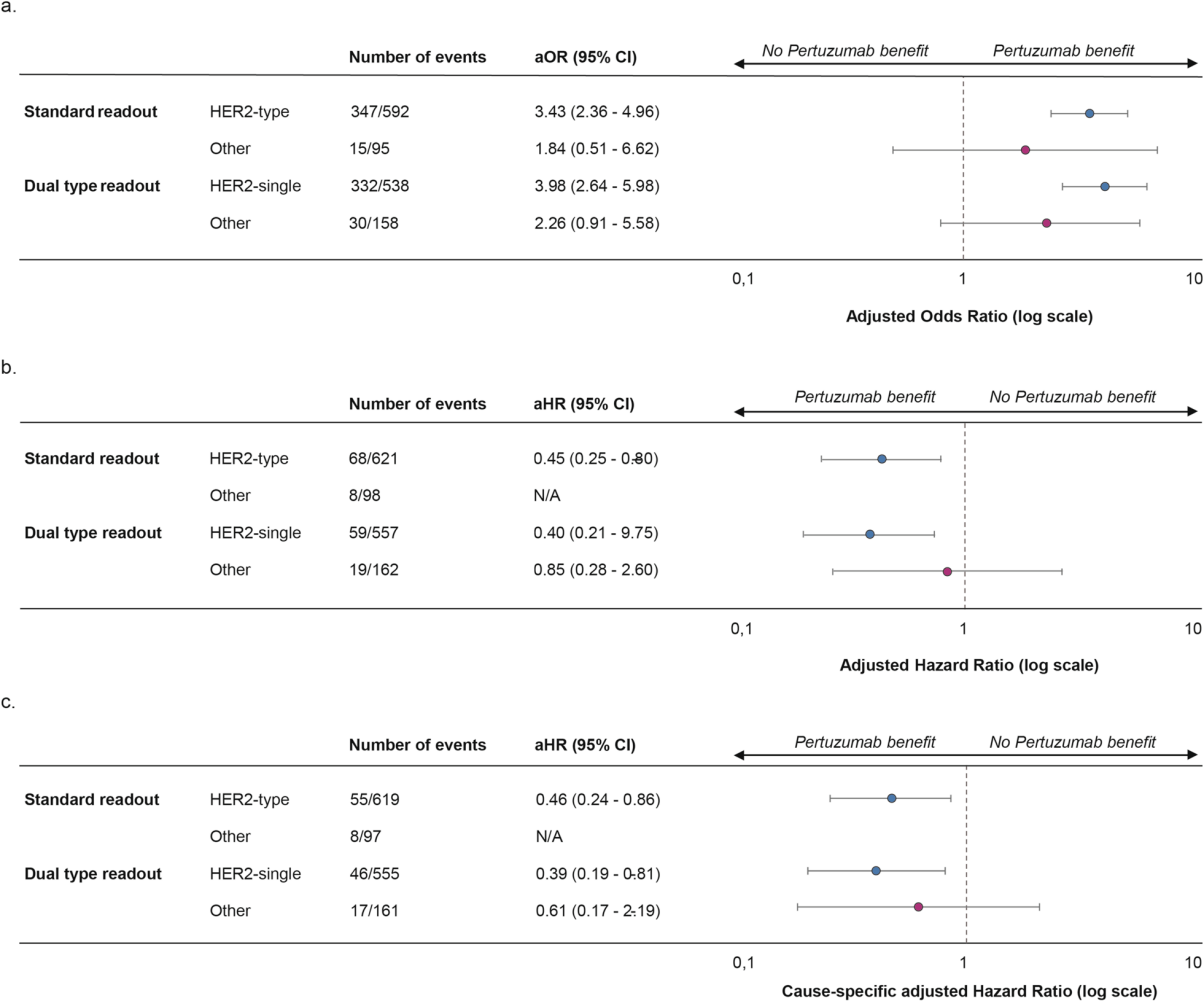
Forest effect of pertuzumab on pathological complete response, overall survival and breast cancer-specific survival per BluePrint standard and dual subtype. **a** Forest plot for pathological complete response. **b** Forest plot for overall survival. The non-HER2 subgroup (“other”) is not plotted due to the low number of events and wide confidence interval. **c**. Forest plot for breast cancer-specific survival. The non-HER2 subgroup (“other”) is not plotted due to the low number of events and wide confidence interval. Error bars represent 95% confidence intervals. *aOR* adjusted Odds Ratio, *aHR* adjusted Hazard Ratio, *95% CI* 95% Confidence Interval, *N/A* not applicable

In a subgroup analysis of patients with HER2-type tumors, the odds of reaching pCR were significantly increased when treated with pertuzumab (aOR 3.43, 95% CI 2.36–4.96, p < 0.001, n = 601; Fig. 4a). No significant beneficial effect of pertuzumab was seen in the patients with a non-HER2-type tumor overall (aOR 1.84, 95% CI 0.51–6.62, p = 0.35, n = 95; Fig. 4a) or within the Luminal-type subgroup (aOR 1.72, 95% CI 0.44–6.73, n = 85). The Basal-type subgroup was deemed too small for subgroup analysis (n = 10).

### Association of BluePrint dual-subtype readout and treatment with pCR

Following dual-subtype classification, a pCR rate of 77.2% was observed in the HER2-single-type tumors that were treated with pertuzumab versus 46.5% in the non-pertuzumab group (Fig. 3b). A significant association of BluePrint dual subtype with pCR was seen in univariable logistic regression analysis (Additional file 2: Table S1). Multivariable regression analysis of BluePrint dual subtype as a binary variable (HER2-single-type versus all other subtypes grouped together, also referred to as non-HER2-single-type) showed a 85% lower odds of reaching pCR for patients with tumors of other subtypes versus the HER2-single-type (Table 2; Additional file 2:Table S2). In addition, analysis of BluePrint dual subtype as a categorical variable showed that presence of the Basal-single-, Luminal-single-, Luminal-HER2- and HER2-Basal-type was negatively associated with pCR (Additional file 2: Tables S1 and S2). No significant interaction between BluePrint dual subtype and HER2 IHC score was seen (p = 0.085). In the IHC 3 + subgroup, a strong negative association was seen for the non-HER2-single-type and pCR (aOR 0.11, 95% CI 0.06–0.20, p < 0.001, median n of pooled model = 633), whereas no such association was seen in patients with IHC 1–2 + tumors (aOR 1.10, 95% CI 0.11–9.03, p = 0.99, median n of pooled model = 63). Additionally, the non-HER2-single subtype was associated with significantly lower odds of reaching pCR in both patients with HR positive (aOR 0.16, 95% CI 0.09–0.27, p < 0.001, median n of pooled model = 439) and patients with HR-negative disease (aOR 0.13, 95% CI 0.03–0.49, p = 0.003, median n of pooled model = 256).

Subgroup analyses showed a significant benefit of pertuzumab in the HER2-single-type subgroup (aOR 3.98, 95% CI 2.64–5.98, p < 0.001, n = 538; Fig. 4a). In the non-HER2-single-type subgroup, the estimated pertuzumab benefit was smaller and not statistically significant, although the group size may limit interpretation (aOR 2.26, 95% CI 0.91–5.58, p = 0.08, n = 158; Fig. 4a).

### Association of BluePrint standard readout and treatment with OS

**Fig. 5:**
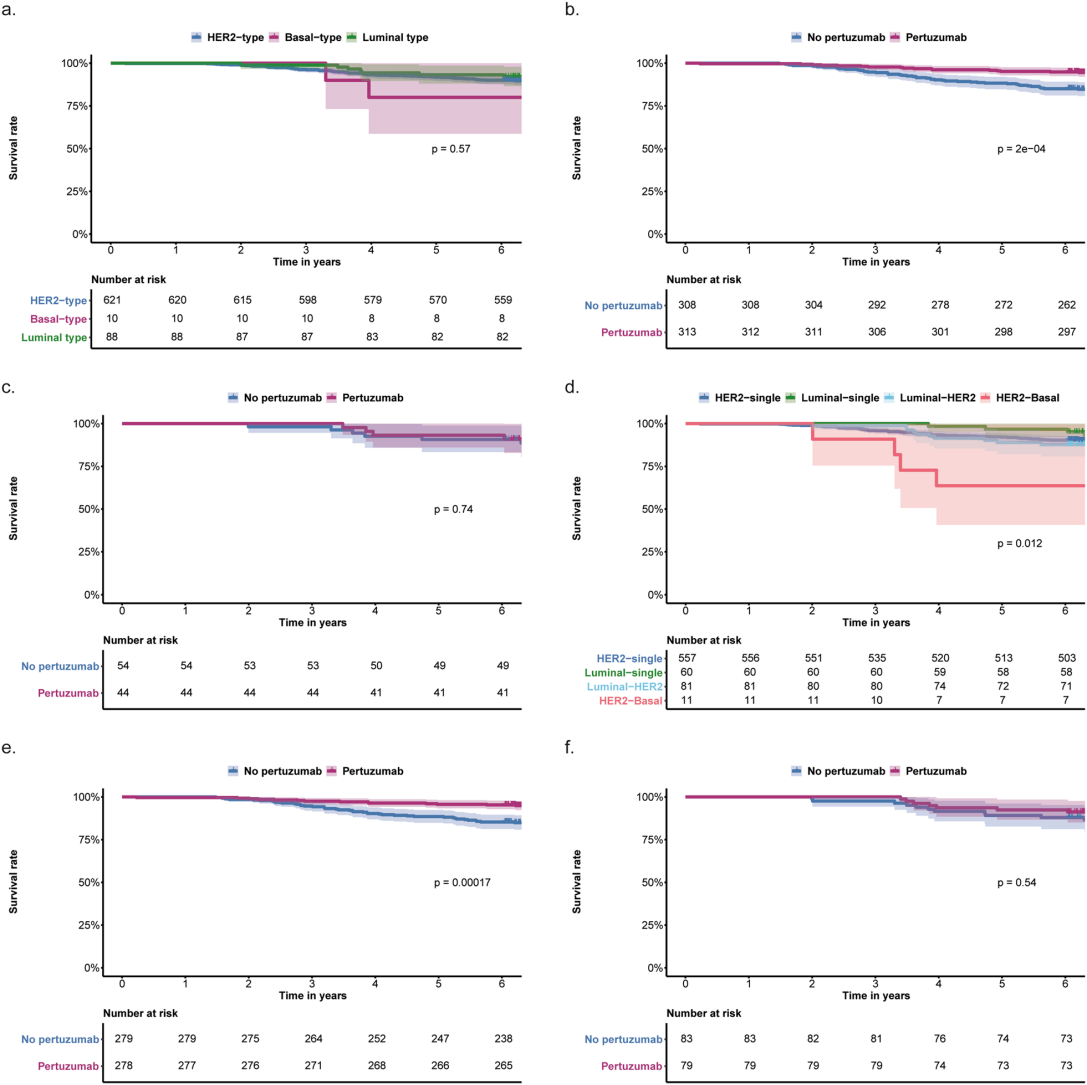
Kaplan–Meier curves of overall survival according to subtype and treatment. **a** Overall survival for the three subtypes according to the BluePrint standard readout, **b** Overall survival according to pertuzumab treatment in the HER2-type determined by BluePrint standard readout, **c** Overall survival according to pertuzumab treatment in other subtypes determined by BluePrint standard readout, **d** Overall survival for the subtypes according to the BluePrint dual-subtype readout. Subtypes with n < 10 are omitted, **e** Overall survival according to pertuzumab treatment in the HER2-single-type determined by BluePrint dual-subtype readout, **f** Overall survival according to pertuzumab treatment in the other subtypes determined by BluePrint dual-subtype readout. Shaded areas represent 95% confidence intervals. All p-values are from log-rank tests

Median follow-up was 6.9 years, during which 78 patients had died. Kaplan–Meier analysis did not show significant differences in OS for the standard BluePrint subtypes (Fig. 5a; Additional file 2: Table S3). BluePrint standard subtypes were not significantly associated with OS in univariable and multivariable analyses (Additional file 2: Table S1; Table 2). No significant interactions between BluePrint subtype and treatment or clinical variables were observed with regard to OS.

In patients with HER2-type tumors who received pertuzumab, 5-year overall survival was 95.2%, compared to 88.3% for patients with HER2-type tumors that did not receive pertuzumab (Fig. 5b; Additional file 2: Table S4). For patients with tumors of other, non-HER2 subtypes, 5-year OS was 93.1% with and 90.7% without pertuzumab (Fig. 5c; Additional file 2: Table S4).

In the HER2-type subgroup, a significant benefit of pertuzumab was seen (adjusted hazard ratio [aHR] 0.45, 95% CI 0.25–0.80, p = 0.007, n = 621; Fig. 4b). The amount of events in the non-HER2-type subgroup was too limited for subgroup analysis (n of events = 8).

### Association of BluePrint dual-subtype readout and treatment with OS

For the different BluePrint dual subtypes a significant difference in OS was observed (only subtypes with n > 10 analyzed; Fig. 5d; Additional file 2: Table S3) in Kaplan–Meier analysis, where patients with a single-Luminal-type tumor had the best 5-year OS (96.7%, 95% CI 92.2–100; Additional file 2: Table S3) and patients with HER2-Basal subtypes had the worst outcome (5-year OS 63.7%, 95% CI 40.7–99.5; Additional file 2: Table S3). Univariable Cox regression analysis of BluePrint dual subtypes demonstrated that Luminal-HER2 and HER2-basal subtypes were significantly associated with worse overall survival compared to the HER2-single-type (Additional file 2: Table S1). When non-HER2-single subtypes were grouped together, a significantly higher risk of an event was observed compared to patients with the HER2-single subtype in multivariable Cox regression (aHR 2.04, 95% CI 1.08–3.84, p = 0.03, n = 719; Table 2; Additional file 2: Table S2). No significant interactions were found between BluePrint dual subtype and treatment or other clinical variables.

Five-year overall survival was 95.7% in patients with HER2-single-type tumors who were treated with pertuzumab versus 88.5% for those who were not (Fig. 5e; Additional file 2: Table S4). In patients with tumors of other subtypes, 5-year OS was 92.4% with pertuzumab and 89.2% without (Fig. 5f; Additional file 2: Table S4). Multivariable Cox regression showed a significant beneficial effect of pertuzumab on OS in HER2-single-type tumors (aHR 0.40, 95% CI 0.210–0.753, p = 0.005, n = 557; Fig. 4b). No such effect was seen in the non-HER2-single-type subgroup (aHR 0.85, 95% CI 0.28–2.60, p = 0.74, n = 162; Fig. 4b).

### Association of BluePrint standard readout and treatment with BCSS

Of the 78 patients who died, 63 died of breast cancer, 12 of other causes, and 3 patients died of unknown cause. Kaplan–Meier analysis did not show a significant difference in BCSS for the different BluePrint standard subtypes (Additional file 1: Figure S2a; Additional file 2: Table S3). No significant association between BluePrint standard subtype and BCSS was seen in both a cause-specific hazard function and a subdistribution hazard model, adjusted for covariables (aHR 1.35, 95% CI 0.54–3.39, p = 0.52, n = 716; Table 2).

In patients with HER2-type tumors who were treated with pertuzumab, BCSS was significantly better than for patients who were not treated with pertuzumab, whereas no such difference was observed in other subtypes (Additional file 1: Figure S2b-c). Subgroup analyses showed a significantly decreased hazard for breast cancer death for patients treated with pertuzumab in the HER2-type subgroup (aHR 0.46, 95% CI 0.4–0.86, p = 0.016, n = 619; Fig. 4c). In the non-HER2 subgroup, the number of patients and events was too low for meaningful analysis (n of events = 8).

### Association of BluePrint dual-subtype readout and treatment with BCSS

A significant difference in BCSS was observed between the BluePrint dual subtypes. Patients with single-Luminal-type tumors showed the best 5-year BCSS (96.7%, 95% CI 92.1–100; Additional file 1: Figure S2d; Additional file 2: Table S3), and patients with HER2-Basal the lowest (63.6, 95% CI 40.7–99.5; Additional file 2: Table S3). In patients with HER2-single-type tumors, BCSS was significantly better for patients treated with pertuzumab (Additional file 1: Figure S2e). No difference in BCSS according to pertuzumab treatment was observed for patients with other tumor subtypes (Additional file 1: Figure S2f). A significant increase was observed in the cause-specific hazard for breast cancer death for patients with a non-HER2-single subtype tumor (aHR 2.38, 95% CI 1.20–4.70, p = 0.014, n = 716; Table 2), which was confirmed in a subdistribution hazard model (p = 0.03).

In a subgroup analysis of the HER2-single-type tumors, pertuzumab treatment was associated with lower hazard of breast cancer death in the presence of competing risks (aHR 0.392, 95% CI 0.19–0.81, p = 0.012, n = 555; Fig. 4c). In the non-HER2-single-type subgroup, no significant benefit of pertuzumab was seen (aHR 0.61, 95% CI 0.17–2.19, p = 0.39, n = 161; Fig. 4c).

## Discussion

The results of our study show that molecular subtypes as determined by BluePrint are associated with response to neoadjuvant pertuzumab in stage II and III HER2-positive breast cancer, independent of other clinical variables such as hormone receptor status and HER2 immunohistochemistry score. Patients with tumors that are classified as HER2-type according to the standard BluePrint or further classified as HER2-single-type according to the dual-subtype readout have a high chance of reaching pathological complete response after neoadjuvant treatment with chemotherapy, trastuzumab and pertuzumab. In addition, a clear benefit of pertuzumab for overall survival and breast cancer-specific survival was seen in the patients with HER2-type or HER2-single-type tumors, which was not seen for patients with other subtypes.

While molecular subtypes have been previously evaluated in HER2-positive breast cancer, we are the first to compare the effects of neoadjuvant chemotherapy combined with trastuzumab and pertuzumab versus trastuzumab only within different molecular subtypes, in relation to both pCR as well as survival outcomes, in such a large sample size. Previously, it was shown in the NBRST study that patients with HER2-type breast tumors had the highest pCR rates, in particular when treated with dual HER2-blockade. However, the study was underpowered for evaluation of long-term survival and treatment interactions were not reported. The study also did not include subgroup analyses and evaluation of the dual subtypes [28]. Recently, results of the APHINITY trial suggested that patients with HER2-single-type tumors might have a greater benefit of adjuvant pertuzumab than patients with other subtypes [16]. Of note, due to the differences in inclusion criteria the population of the APHINITY trial may have a different prognosis than our population. In addition, subtype evaluation in the APHINITY study was performed in a nested case–control set, resulting in a much higher proportion of Luminal- and Basal-type tumors compared to our study. Therefore, associations were analyzed by inverse probability weight corrected Cox regression, which showed a trend toward greater pertuzumab benefit in the patients with HER2-type tumors, similar to our findings. Additionally, a systematic review analyzing 16 studies in early-stage HER2-positive disease for which PAM50 subtyping was performed, found that the HER2-enriched subtype was significantly associated with pathological complete response independent of hormone receptor status [13]. The effect of dual HER2-blockade versus single HER2-blockade within different subtypes was not evaluated, and long-term outcomes were not assessed. The design and sample size of our study allowed us to not only evaluate molecular subtypes in relation to both overall as well as breast cancers specific subtypes, but also study the effect of dual versus single HER2-blockade in relation to molecular subtypes through interaction tests and subgroup analyses. However, given that the majority of HER2-positive tumors in our study population exhibits a HER2-activated subtype, other subtypes were grouped together in most analyses. The absence of a significant interaction in our study may indicate that molecular subtype is not specific to pertuzumab benefit. Indeed, molecular subtype seems predictive of response to HER2-targeting therapy in general, since the patients who received single HER2-blockade treatment with trastuzumab only and have a HER2-type tumor also have better pCR rates than patients with other tumor subtypes. However, since we did observe a clear benefit of additional treatment with pertuzumab in HER2-type tumors and not in non-HER2-type tumors based on subgroup analyses, the lack of interaction may also be due to the smaller sample size of the non-HER2-type group. Further studies are warranted to validate our findings and confirm the lack of pertuzumab benefit in patients with clinically HER2-positive but genomically non-HER2-type tumors. Given that dual HER2-blockade is currently standard of care for patients with stage II-III HER2-positive breast cancer, this could be analyzed by extending the current analyses to include patients treated after 2016.

HER2-targeting agents are known to exert their effect by both blocking the HER2-pathway and through activation of natural killer cell-mediated antibody-dependent cellular cytotoxicity (ADCC) [29, 30]. Results of several clinical trials indicate that a subgroup of patients responds well to treatment with only dual HER2-blockade, without chemotherapy [4, 9–11]. It is hypothesized that these patients’ tumors rely heavily on the HER2-pathway for survival and thus might be particularly sensitive to the combination of HER2-pathway blockade and ADCC. Finding biomarkers that identify this group of patients with ‘HER2-driven’ tumors is highly relevant for future studies evaluating de-escalation of chemotherapy. Given that BluePrint was developed through supervised analysis based on ER, PR and HER2 status (IHC and mRNA expression), it might be able to capture subtype-specific pathways better than previously used methods, such as PAM50 subtyping [14, 31]. In addition, the recently developed BluePrint dual-subtype readout has shown that some tumors display multiple activated pathways and appear to be biologically different from the true single-subtype tumors, which may be valuable for further distinction of truly HER2-driven tumors and relevant for the probability of response to HER2-targeted treatment [16, 24, 25]. Our results show that the BluePrint HER2-single-subtype indeed seems to be a stronger prognostic factor than the standard readout HER2-type. Many patients that were initially classified as HER2-type by the standard readout are classified as Luminal-HER2-type upon dual readout, which is associated with lower odds of pCR and worse prognosis in both univariable and multivariable analyses. Interestingly, we found that among patients with a high HER2 immunohistochemistry score presence of a subtype other than HER2 or HER2-single is associated with a severely diminished chance of reaching pathological complete response. This suggests that HER2 IHC scoring may not fully account for tumor heterogeneity and that molecular subtyping may have additional value to HER2 IHC scoring for prediction of pCR after neoadjuvant treatment with chemotherapy and HER2-blockade. Thus, BluePrint molecular subtypes may be valuable in conjunction to other biomarkers to identify HER2-driven tumors. Since HER2 IHC scoring was not performed centrally and that detailed information on HER2 evaluation was not available for further analysis, it cannot be ruled out some tumors may have been falsely classified as HER2 positive. In addition, we found the non-HER2-single subtype to be associated with a lower chance of pCR in both hormone receptor-negative and hormone receptor-positive diseases. However, the heterogeneity of molecular subtypes is substantially smaller among patients with hormone receptor-negative breast cancer, and further research is needed to investigate clinical utility of subtyping in this group of patients.

Given that HER2-type and HER2-single-type tumors had excellent prognosis after treatment with trastuzumab and pertuzumab, molecular subtyping may be informative for the selection of patients who could be potential candidates for treatment de-escalation. The TRAIN-3 study is a de-escalation trial in which patients with stage II-III HER2-positive disease are referred to surgery once they reach pCR during neoadjuvant treatment with chemotherapy and dual HER2-blockade; our data may be validated by retrospective analysis of molecular subtype in these patients. In addition, our results could be confirmed further in future trials by randomizing patients with genomically HER2-type tumors between standard treatment with dual neoadjuvant chemotherapy and dual HER2-blockade, versus treatment with dual HER2-blockade only. Besides its potential use in de-escalation, molecular subtyping may identify patients with low chances of response or poor prognosis, who could benefit of treatment strategies other than HER2-blockade. Current adjuvant treatment decisions are based on presence of pCR following neoadjuvant treatment, where patients with no pCR are treated with trastuzumab-emtansine (T-DM1) [32, 33]. Results of recent studies indicate that trastuzumab-deruxtecan (T-DXd) may benefit both patients with low HER2-expression and HER2-positive metastatic patients who acquired resistance to T-DM1 [34, 35]. Given that T-DXd is thought to have a more potent bystander killing effect than T-DM1 by penetrating cells adjacent to HER2-positive cells, it may be a highly interesting treatment option in the early-stage setting for patients with tumors that are HER2 positive based on immunohistochemistry (score of 3 +), but of a non-HER2 subtype as determined by gene expression analysis.

Our study has a few limitations. Despite taking measures to ensure adequate matching, cases and controls differed with respect to chemotherapy regimens, tumor grade and nodal stage. Given that anthracycline-based treatment was preferred as a chemotherapy regimen in the Netherlands during the study period, the majority of control patients had received anthracyclines, which made perfect matching impossible, and also affected the matching of the other clinical variables [36]. In addition, tumor grade was only available after case–control selection and thus not accounted for during matching. Tumor stage (II vs. III) was included as a matching factor but could not prevent an imbalance in nodal stage. Given that several studies have shown that anthracycline-containing and anthracycline-free chemotherapy regimens lead to comparable outcomes in HER2-positive breast cancer, we consider it unlikely that this has substantially affected our results [6, 8, 17, 37]. In addition, since the imbalances are not severe and all analyses have been corrected for these variables, we are confident that they have not impacted our results significantly, although residual confounding cannot be fully excluded. The majority of patients that received pertuzumab underwent their treatment as part of the TRAIN-2 clinical trial, whereas patients that did not receive pertuzumab were not trial participants. Given that our study is based on data from the Netherlands Cancer Registry, which does not provide detailed information on comorbidity, we cannot fully exclude the possibility that the non-pertuzumab cohort is overall less healthy than the pertuzumab cohort. However, since some hospitals did and some did not participate in the TRAIN-2 trial, and inclusion rates were high among participating hospitals, study participation was largely based on in which hospital the diagnosis was made and thus mostly arbitrary. In addition, since all of the patients in our study cohort were fit for chemotherapy and were of a similar age, we have no reason to assume substantial health differences between the pertuzumab and the non-pertuzumab groups. Due to the imbalances in the matched groups and the potential selection bias, further validation of our results in independent datasets is warranted.

In conclusion, our results indicate that in patients with stage II-III HER2-positive breast tumors that are classified as HER2-type or HER2-single-type upon molecular characterization, the addition of pertuzumab to neoadjuvant chemotherapy and trastuzumab may improve pathological complete response and may decrease the risk of death due to breast cancer. Given the excellent long-term outcomes after treatment with dual HER2-blockade in patients with HER2-type or HER2-single-type tumors, molecular subtyping might be a valuable biomarker for candidate selection in future trials investigating either de-escalation of neoadjuvant chemotherapy, or alternative or intensified treatment strategies. Prospective validation of our findings is needed to confirm the role of BluePrint in patient selection for dual HER2-blockade.

## Supplementary Information

**Additional file 1.** Supplementary Figures.

**Additional file 2.** Supplementary Tables.

## Data Availability

The data sets generated and/or analyzed during the current study are not publicly available, as the study has used external data from the Netherlands Cancer Registry which was then linked with data from PALGA and Statistics Netherlands. The data sets will be made available from the Netherlands Cancer Registry upon reasonable request (data request study number K16.130). To apply for data access, please visit https://www.iknl.nl/en/ncr/apply-for-data. The raw microarray data for this study were generated at Agendia NV. The microarray subtype readouts generated in this study are not publicly available due to patient privacy but are available upon reasonable request from the corresponding author with the permission of Agendia NV.

## References

[1] Gianni L, Pienkowski T, Im YH, Roman L, Tseng LM, Liu MC (2012). Efficacy and safety of neoadjuvant pertuzumab and trastuzumab in women with locally advanced, inflammatory, or early HER2-positive breast cancer (NeoSphere): a randomised multicentre, open-label, phase 2 trial. Lancet Oncol.

[2] Schneeweiss A, Chia S, Hickish T, Harvey V, Eniu A, Hegg R (2013). Pertuzumab plus trastuzumab in combination with standard neoadjuvant anthracycline-containing and anthracycline-free chemotherapy regimens in patients with HER2-positive early breast cancer: a randomized phase II cardiac safety study (TRYPHAENA). Ann Oncol.

[3] van Ramshorst MS, van der Voort A, van Werkhoven ED, Mandjes IA, Kemper I, Dezentje VO (2018). Neoadjuvant chemotherapy with or without anthracyclines in the presence of dual HER2 blockade for HER2-positive breast cancer (TRAIN-2): a multicentre, open-label, randomised, phase 3 trial. Lancet Oncol.

[4] Gianni L, Pienkowski T, Im YH, Tseng LM, Liu MC, Lluch A (2016). 5-year analysis of neoadjuvant pertuzumab and trastuzumab in patients with locally advanced, inflammatory, or early-stage HER2-positive breast cancer (NeoSphere): a multicentre, open-label, phase 2 randomised trial. Lancet Oncol.

[5] von Minckwitz G, Procter M, de Azambuja E, Zardavas D, Benyunes M, Viale G (2017). Adjuvant pertuzumab and trastuzumab in early HER2-positive breast cancer. N Engl J Med.

[6] Schneeweiss A, Chia S, Hickish T, Harvey V, Eniu A, Waldron-Lynch M (2018). Long-term efficacy analysis of the randomised, phase II TRYPHAENA cardiac safety study: evaluating pertuzumab and trastuzumab plus standard neoadjuvant anthracycline-containing and anthracycline-free chemotherapy regimens in patients with HER2-positive early breast cancer. Eur J Cancer.

[7] Garrison LP, Babigumira J, Tournier C, Goertz HP, Lubinga SJ, Perez EA (2019). Cost-effectiveness analysis of pertuzumab with trastuzumab and chemotherapy compared to trastuzumab and chemotherapy in the adjuvant treatment of HER2-positive breast cancer in the United States. Value Health.

[8] Piccart M, Procter M, Fumagalli D, de Azambuja E, Clark E, Ewer MS (2021). Adjuvant Pertuzumab and Trastuzumab in Early HER2-Positive Breast Cancer in the APHINITY Trial: 6 Years' Follow-Up. J Clin Oncol.

[9] Perez-Garcia JM, Gebhart G, Ruiz Borrego M, Stradella A, Bermejo B, Schmid P (2021). Chemotherapy de-escalation using an (18)F-FDG-PET-based pathological response-adapted strategy in patients with HER2-positive early breast cancer (PHERGain): a multicentre, randomised, open-label, non-comparative, phase 2 trial. Lancet Oncol.

[10] Connolly RM, Leal JP, Solnes L, Huang CY, Carpenter A, Gaffney K (2021). Updated results of TBCRC026: phase II trial correlating standardized uptake value with pathological complete response to pertuzumab and trastuzumab in breast cancer. J Clin Oncol.

[11] Gluz O, Nitz U, Christgen M, Kuemmel S, Holtschmidt J, Priel J, et al. De-escalated chemotherapy versus endocrine therapy plus pertuzumab+ trastuzumab for HR+/HER2+ early breast cancer (BC): First efficacy results from the neoadjuvant WSG-TP-II study. Journal of Clinical Oncology. 2020;38(15_suppl):515.

[12] Veeraraghavan J, De Angelis C, Reis-Filho JS, Pascual T, Prat A, Rimawi MF (2017). De-escalation of treatment in HER2-positive breast cancer: determinants of response and mechanisms of resistance. Breast.

[13] Schettini F, Pascual T, Conte B, Chic N, Braso-Maristany F, Galvan P (2020). HER2-enriched subtype and pathological complete response in HER2-positive breast cancer: a systematic review and meta-analysis. Cancer Treat Rev.

[14] Krijgsman O, Roepman P, Zwart W, Carroll JS, Tian S, de Snoo FA (2012). A diagnostic gene profile for molecular subtyping of breast cancer associated with treatment response. Breast Cancer Res Treat.

[15] Kuilman MM, Ellappalayam A, Barcaru A, Haan JC, Bhaskaran R, Wehkamp D (2022). BluePrint breast cancer molecular subtyping recognizes single and dual subtype tumors with implications for therapeutic guidance. Breast Cancer Res Treat.

[16] Krop I, Mittempergher L, Paulson J, Andre F, Bonnefoi H, Loi S, et al. Abstract PD3-01: BluePrint performance in predicting pertuzumab benefit in genomically HER2-positive patients: A biomarker analysis of the APHINITY trial. Cancer Res. 2021; 81(4 Suppl):PD3-01-PD3.

[17] van der Voort A, van Ramshorst MS, van Werkhoven ED, Mandjes IA, Kemper I, Vulink AJ (2021). Three-year follow-up of neoadjuvant chemotherapy with or without anthracyclines in the presence of dual ERBB2 blockade in patients with ERBB2-positive breast cancer: a secondary analysis of the TRAIN-2 randomized, phase 3 trial. JAMA Oncol.

[18] van Ramshorst MS, van Werkhoven E, Honkoop AH, Dezentje VO, Oving IM, Mandjes IA (2016). Toxicity of dual HER2-blockade with pertuzumab added to anthracycline versus non-anthracycline containing chemotherapy as neoadjuvant treatment in HER2-positive breast cancer: The TRAIN-2 study. Breast.

[19] Casparie M, Tiebosch AT, Burger G, Blauwgeers H, van de Pol A, van Krieken JH (2007). Pathology databanking and biobanking in The Netherlands, a central role for PALGA, the nationwide histopathology and cytopathology data network and archive. Cell Oncol.

[20] Slide Score. Available from: https://www.slidescore.com.

[21] Nationaal Borstkanker Overleg Nederland. Dutch Guideline Breast Cancer 2012 [Available from: https://richtlijnendatabase.nl/richtlijn/borstkanker/algemeen.html.

[22] Wolff AC, Hammond ME, Hicks DG, Dowsett M, McShane LM, Allison KH (2013). Recommendations for human epidermal growth factor receptor 2 testing in breast cancer: American Society of Clinical Oncology/College of American Pathologists clinical practice guideline update. J Clin Oncol.

[23] Ho D, Imai K, King G, Stuart EA (2011). MatchIt: Nonparametric Preprocessing for Parametric Causal Inference. J Stat Softw.

[24] Mittempergher L, Delahaye LJ, Witteveen AT, Snel MH, Mee S, Chan BY (2020). Performance characteristics of the BluePrint(R) breast cancer diagnostic test. Transl Oncol.

[25] Ellappalayam A, Kuilman M, Mittempergher L, Wehkamp D, Chan B, Bhaskaran R (2020). 518 Poster–BluePrint molecular subtyping recognizes single and dual subtype tumors with consequences for therapeutic guidance. Eur J Cancer.

[26] van Buuren S, Groothuis-Oudshoorn K (2011). mice: Multivariate Imputation by Chained Equations in R. J Stat Softw.

[27] R Core Team. R: A language and environment for statistical computing: R Foundation for Statistical Computing, Vienna, Austria.; 2022 https://www.R-project.org/.

[28] Whitworth PW, Beitsch PD, Murray MK, Richards PD, Mislowsky A, Dul CL (2022). Genomic classification of HER2-positive patients with 80-gene and 70-gene signatures identifies diversity in clinical outcomes With HER2-targeted neoadjuvant therapy. JCO Precis Oncol.

[29] Scheuer W, Friess T, Burtscher H, Bossenmaier B, Endl J, Hasmann M (2009). Strongly enhanced antitumor activity of trastuzumab and pertuzumab combination treatment on HER2-positive human xenograft tumor models. Cancer Res.

[30] Toth G, Szoor A, Simon L, Yarden Y, Szollosi J, Vereb G (2016). The combination of trastuzumab and pertuzumab administered at approved doses may delay development of trastuzumab resistance by additively enhancing antibody-dependent cell-mediated cytotoxicity. MAbs.

[31] Parker JS, Mullins M, Cheang MC, Leung S, Voduc D, Vickery T (2009). Supervised risk predictor of breast cancer based on intrinsic subtypes. J Clin Oncol.

[32] von Minckwitz G, Huang CS, Mano MS, Loibl S, Mamounas EP, Untch M (2019). Trastuzumab emtansine for residual invasive HER2-positive breast cancer. N Engl J Med.

[33] Cardoso F, Kyriakides S, Ohno S, Penault-Llorca F, Poortmans P, Rubio IT (2019). Early breast cancer: ESMO clinical practice guidelines for diagnosis, treatment and follow-up. Ann Oncol.

[34] Modi S, Saura C, Yamashita T, Park YH, Kim SB, Tamura K (2020). Trastuzumab deruxtecan in previously treated HER2-positive breast cancer. N Engl J Med.

[35] Modi S, Jacot W, Yamashita T, Sohn J, Vidal M, Tokunaga E, et al. Trastuzumab Deruxtecan in Previously Treated HER2-Low Advanced Breast Cancer. N Engl J Med. 2022. 10.1056/NEJMoa2203690 PMC10561652 35665782

[36] van der Voort A, Liefaard MC, van Ramshorst MS, van Werkhoven E, Sanders J, Wesseling J (2022). Efficacy of neoadjuvant treatment with or without pertuzumab in patients with stage II and III HER2-positive breast cancer: a nationwide cohort analysis of pathologic response and 5-year survival. The Breast.

[37] Slamon D, Eiermann W, Robert N, Pienkowski T, Martin M, Press M (2011). Adjuvant trastuzumab in HER2-positive breast cancer. N Engl J Med.

