# Supplementary material for: BluePrint molecular subtypes predict response to neoadjuvant pertuzumab in HER2-positive breast cancer
Source: Breast Cancer Res. 2023 Jun 19;25:71. doi: 10.1186/s13058-023-01664-x (PMC10280902; doi:10.1186/s13058-023-01664-x)
Supplement: Supplementary file 1 — Additional file 1. Supplementary Figures. [file 13058_2023_1664_MOESM1_ESM.docx]

**SUPPLEMENTARY FIGURES**

**Figure S1. BluePrint dual subtypes in the total study population and according to clinical subgroups**

*a. Total group, b. Hormone receptor negative disease, c. Hormone receptor positive disease, d. Node-negative disease, e. Node-positive disease, f. HER2 immunohistochemistry 1-2+ tumors, g. HER2 immunohistochemistry 3+ tumors*

*Abbreviations: HR = hormone receptor; cN0 = clinically node-negative; cN+ = clinically node-positive; HER2 IHC = HER2 immunohistochemistry score*

**
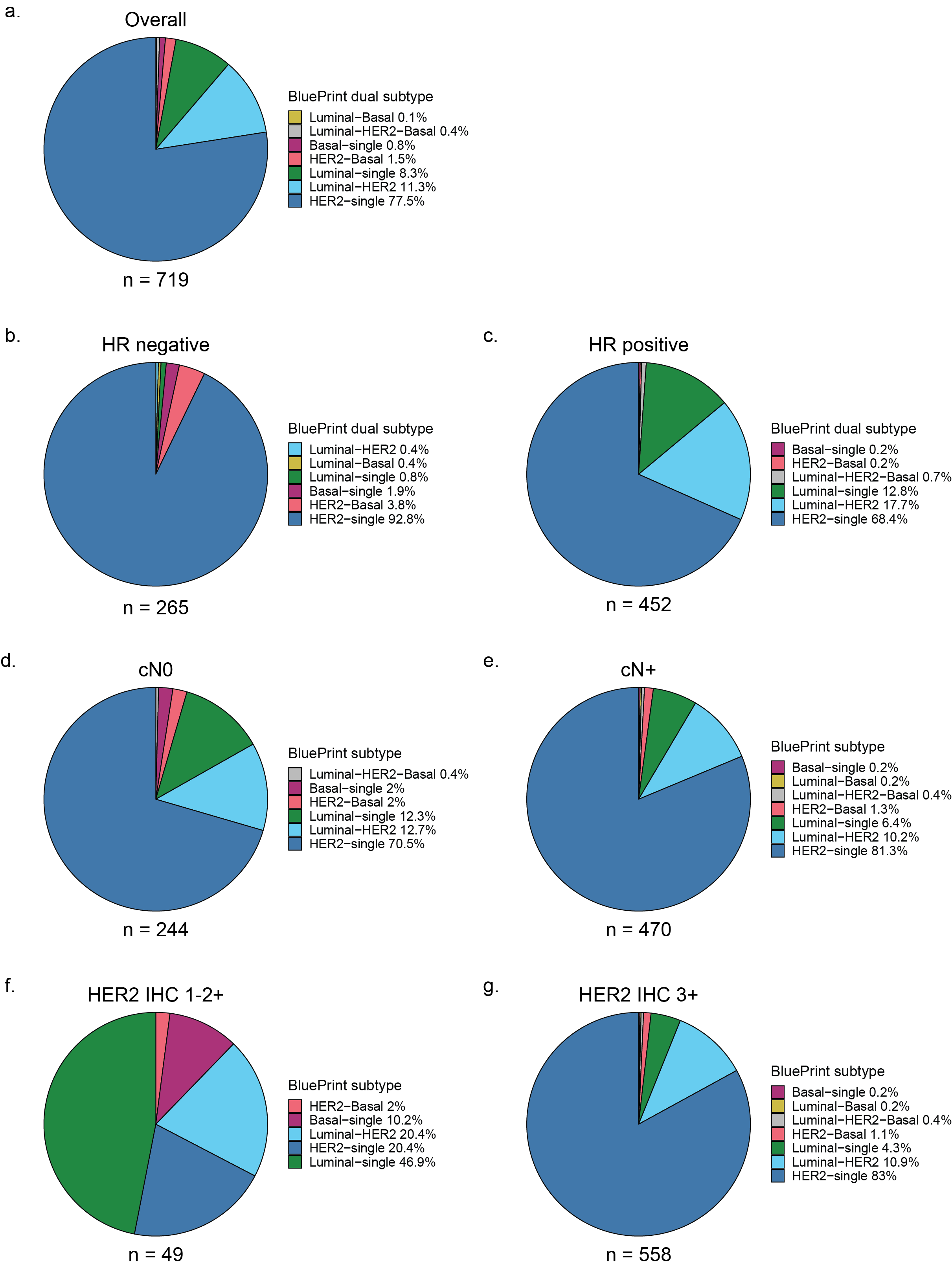
**

**Figure S2. Kaplan-Meier curves of breast cancer specific survival according to subtype and treatment**

*a. Breast cancer specific survival for the three subtypes according to the standard readout, b. Breast cancer specific survival according to pertuzumab treatment in the HER2-type determined by standard readout, c. Breast cancer specific survival according to pertuzumab treatment in other subtypes determined by standard readout, d. Breast cancer specific survival for the subtypes according to the dual subtype readout. Subtypes with n < 10 are omitted. e. Breast cancer specific survival according to pertuzumab treatment in the single HER2-type determined by dual subtype readout, f. Breast cancer specific survival according to pertuzumab treatment in the other subtypes determined by dual subtype readout*

a.


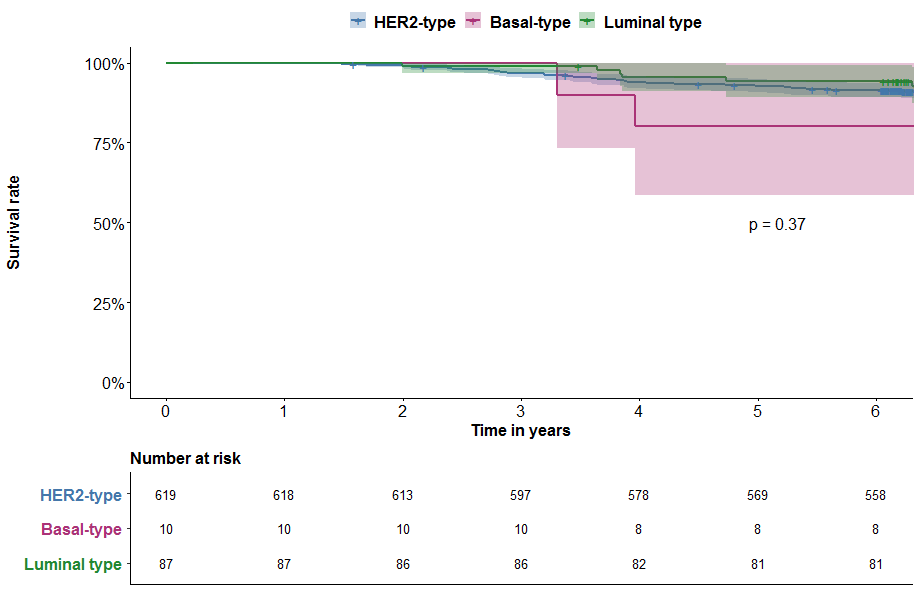


b.

*
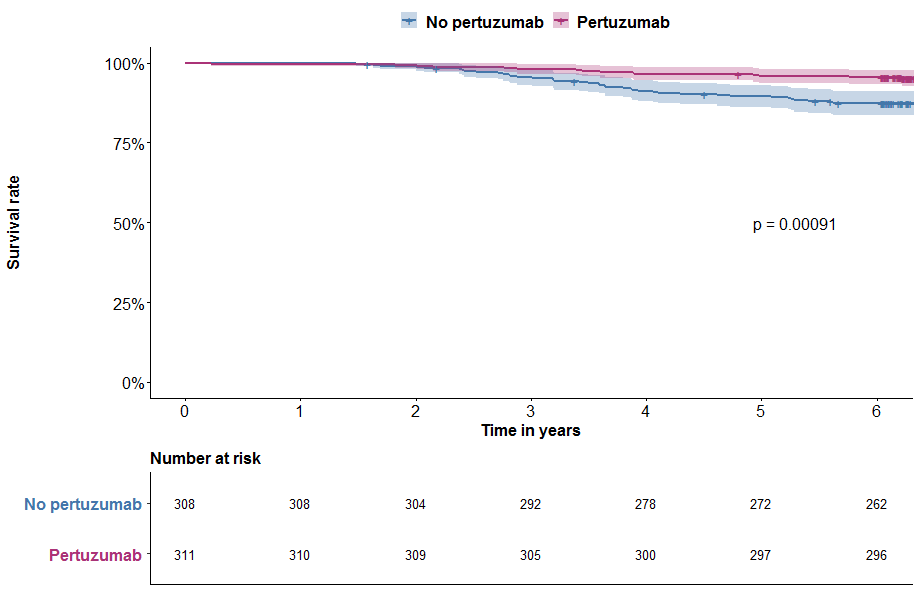
*

c.


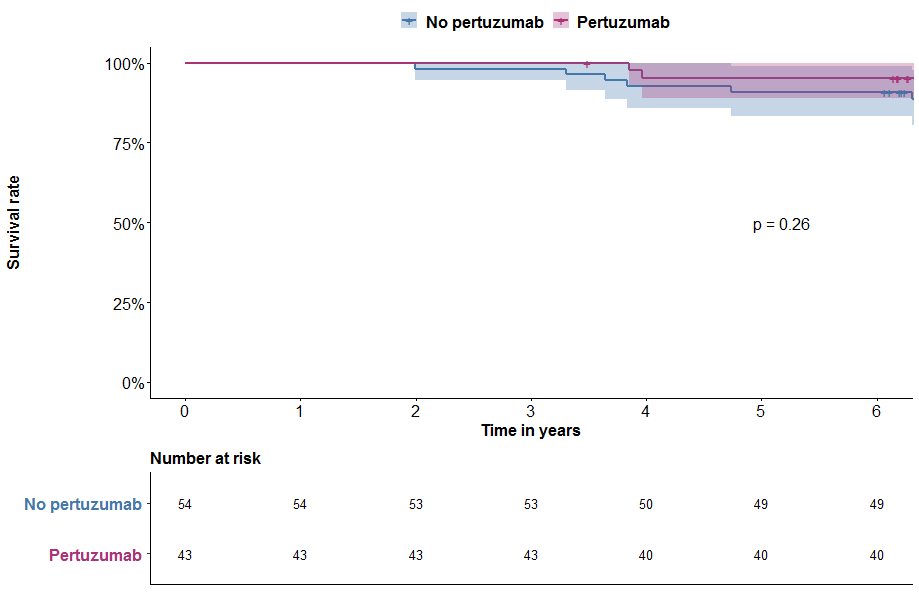


d.


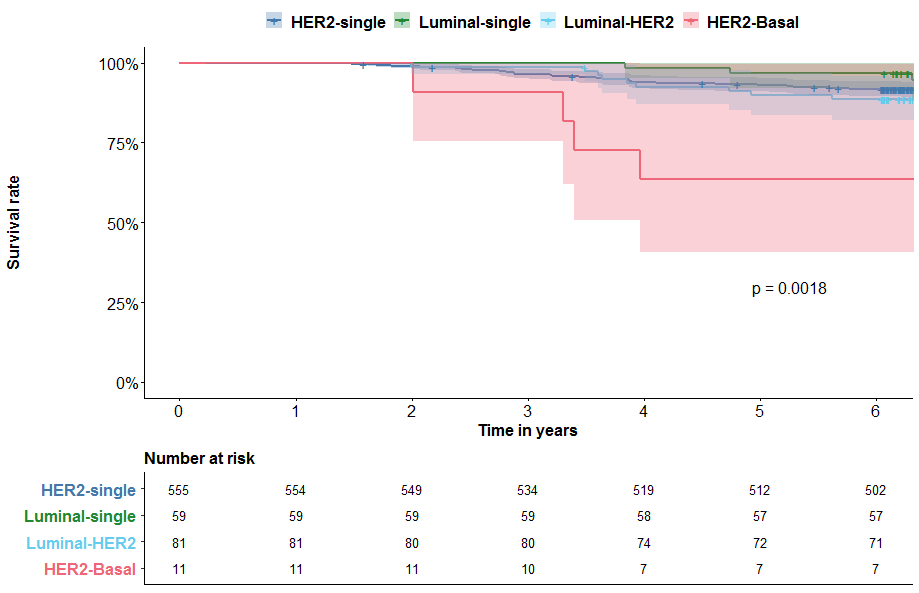


e.


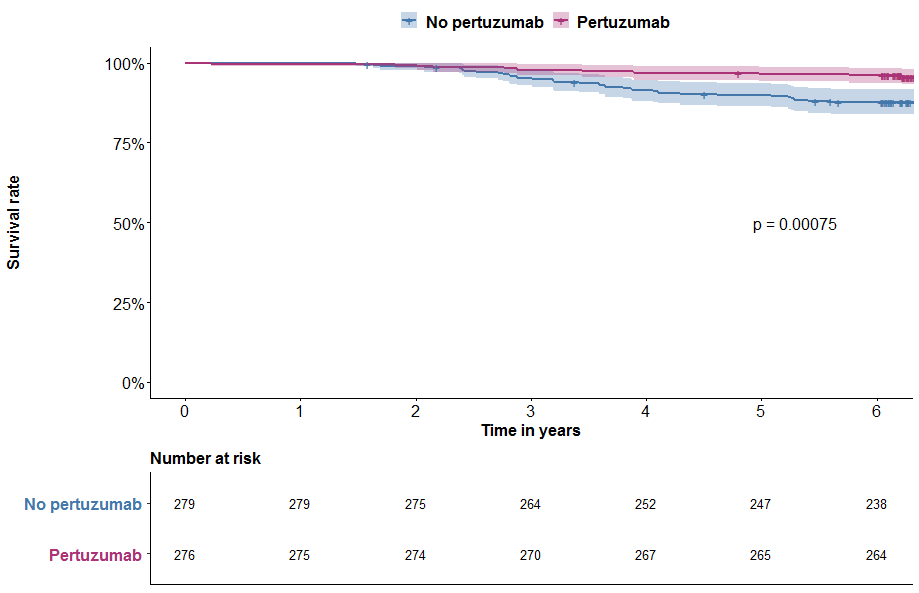


f.

*
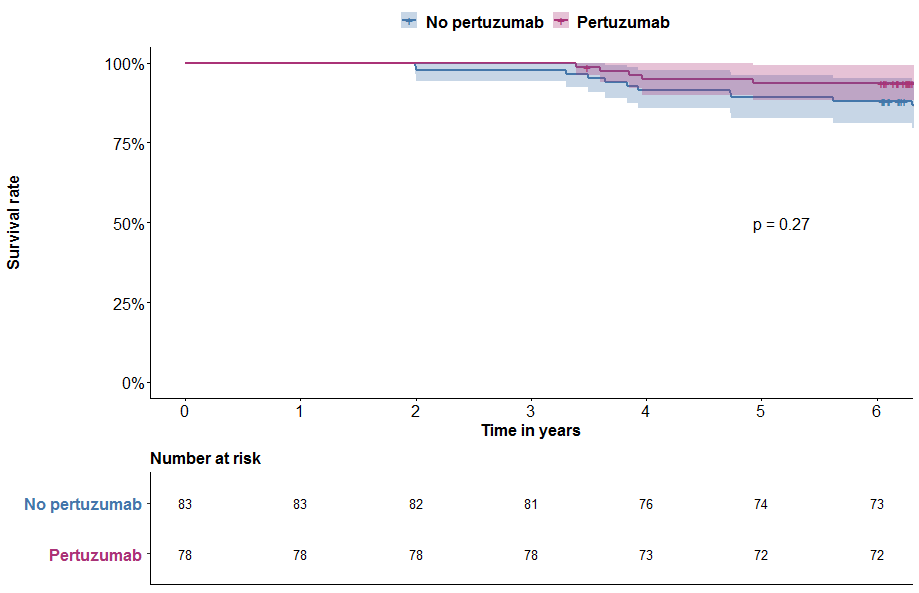
*
