# Supplementary material for: BluePrint molecular subtypes predict response to neoadjuvant pertuzumab in HER2-positive breast cancer
Source: Breast Cancer Res. 2023 Jun 19;25:71. doi: 10.1186/s13058-023-01664-x (PMC10280902; doi:10.1186/s13058-023-01664-x)
Supplement: Supplementary file 2 — Additional file 2. Supplementary Tables. [file 13058_2023_1664_MOESM2_ESM.docx]

**SUPPLEMENTARY TABLES**

**Supplementary Table 1. Univariable logistic and Cox regression analyses of clinical variables and BluePrint subtypes in relation to pCR and OS**

|  | **pCR** | | | **OS** | | |
| --- | --- | --- | --- | --- | --- | --- |
|  | **OR** | **95% CI** | **p** | **HR** | **95% CI** | **p** |
| **Age** | 1.00 | 1.00 – 1.00 | 0.759 | 1.05 | 1.03 – 1.07 | <0.001 |
| **ER status** |  |  |  |  |  |  |
| Negative | Ref | Ref |  | Ref | Ref |  |
| Positive | 0.76 | 0.70 – 0.82 | <0.001 | 0.53 | 0.33 – 0.83 | 0.006 |
| **PR status** |  |  |  |  |  |  |
| Negative | Ref | Ref |  | Ref | Ref |  |
| Positive | 0.74 | 0.68 – 0.79 | <0.001 | 0.79 | 0.49 – 1.25 | 0.306 |
| **HR status** |  |  |  |  |  |  |
| Negative | Ref | Ref |  | Ref | Ref |  |
| Positive | 0.75 | 0.70 – 0.81 | <0.001 | 0.56 | 0.35 – 0.87 | 0.012 |
| **cT** |  |  |  |  |  |  |
| T0–2 | Ref | Ref |  | Ref | Ref |  |
| T3–4 | 0.96 | 0.88 – 1.04 | 0.280 | 2.67 | 1.70 – 4.19 | <0.001 |
| **cN** |  |  |  |  |  |  |
| Negative | Ref | Ref |  | Ref | Ref |  |
| Positive | 0.89 | 0.82 – 0.96 | 0.004 | 5.46 | 2.48 – 12.0 | <0.001 |
| **Grade** |  |  |  |  |  |  |
| 1–2 | Ref | Ref |  | Ref | Ref |  |
| 3 | 1.04 | 0.97 – 1.13 | 0.254 | 1.52 | 0.94 – 2.45 | 0.086 |
| **HER2 IHC** |  |  |  |  |  |  |
| 0–2+ | Ref | Ref |  | Ref | Ref |  |
| 3+ | 1.38 | 1.20 – 1.59 | <0.001 | 1.16 | 0.46 – 2.94 | 0.752 |
| **Anthracyclines** |  |  |  |  |  |  |
| No | Ref | Ref |  | Ref | Ref |  |
| Yes | 0.96 | 0.89 – 1.03 | 0.249 | 0.71 | 0.45 – 1.12 | 0.139 |
| **Pertuzumab** |  |  |  |  |  |  |
| No | Ref | Ref |  | Ref | Ref |  |
| Yes | 1.30 | 1.21 – 1.40 | <0.001 | 0.42 | 0.26 – 0.69 | <0.001 |
| **BluePrint standard subtype (categorical)** |  |  |  |  |  |  |
| HER2–type | Ref | Ref |  | Ref | Ref |  |
| Basal–type | 0.69 | 0.51 – 0.93 | 0.014 | 0.18 | 0.44 – 7.69 | 0.398 |
| Luminal–type | 0.65 | 0.59 – 0.73 | <0.001 | 0.81 | 0.39 – 1.71 | 0.578 |
| **BluePrint standard subtype (binary)** |  |  |  |  |  |  |
| HER2–type | Ref | Ref |  | Ref | Ref |  |
| Non–HER2–type | 0.66 | 0.59 – 0.73 | <0.001 | 0.91 | 0.465 – 1.79 | 0.791 |
| **BluePrint dual subtype (categorical)** |  |  |  |  |  |  |
| Single HER2 | Ref | Ref |  | Ref | Ref |  |
| Single Basal | 0.64 | 0.44 – 0.93 | 0.019 | 1.83e-07 | 0.00 – Inf | 0.996 |
| Single Luminal | 0.63 | 0.55 – 0.71 | <0.001 | 0.60 | 0.21 – 1.68 | 0.327 |
| Luminal–HER2 | 0.68 | 0.61 – 0.76 | <0.001 | 1.31 | 0.68 – 2.52 | 0.412 |
| HER2–Basal | 0.66 | 0.49 – 0.88 | <0.001 | 4.15 | 1.48 – 11.6 | 0.008 |
| Luminal–Basal | 0.54 | 0.22 – 1.35 | 0.188 | 1.82e-07 | 0.00 – Inf | 0.998 |
| Luminal–HER2–Basal | 0.54 | 0.32 – 0.92 | 0.023 | 1.83e-07 | 0.00 – Inf | 0.997 |
| **BluePrint dual subtype (binary)** |  |  |  |  |  |  |
| Single HER2 | Ref | Ref |  | Ref | Ref |  |
| Non–single HER2 | 0.65 | 0.60– 0.71 | <0.001 | 1.11 | 0.65 – 1.87 | 0.703 |

*Abbreviations: pCR = pathological complete response; OS = overall survival; OR = Odds Ratio; 95% CI = 95% Confidence Interval; p = p–value; HR = hazard ratio; Ref = reference; ER status = estrogen receptor status; PR status = progesterone receptor status; HR status = hormone receptor status; cT = clinical T–stage; cN = clinical N–stage; HER2 IHC = HER2 immunohistochemistry score;*

**Supplementary Table 2. All BluePrint standard and dual subtypes in relation to pathological complete response and survival outcomes**

|  |  | **Pathological complete response** | |  | **Overall survival** | |  | **Breast cancer specific survival** | |
| --- | --- | --- | --- | --- | --- | --- | --- | --- | --- |
|  |  | **aOR (95% CI)** | **p** |  | **aHR (95% CI)** | **p** |  | **aHR (95% CI)** | **p** |
| **Standard subtype** | HER2 | Ref |  |  | Ref |  |  | Ref |  |
|  | Basal | 0.11 (0.02 – 0.63) | 0.013 |  | 2.67 (0.47 – 15.02) | 0.26 |  | 3.96 (0.67 – 23.29) | 0.13 |
|  | Luminal | 0.18 (0.09 – 0.35) | <0.001 |  | 1.22 (0.51 – 2.90) | 0.65 |  | 1.16 (0.43 – 3.12) | 0.77 |
|  | All non–HER2 | 0.17 (0.09 – 0.32) | <0.001 |  | 1.33 (0.58 – 3.03) | 0.50 |  | 1.35 (0.54 – 3.39) | 0.52 |
| **Dual subtype** | HER2–single | Ref |  |  | Ref |  |  | Ref |  |
|  | Basal–single | 0.07 (0.01 – 0.77) | 0.03 |  | N/A |  |  | N/A |  |
|  | Luminal–single | 0.13 (0.01 – 0.29) | <0.001 |  | 1.08 (0.34– 3.42) | 0.90 |  | 1.12 (0.29 – 4.23) | 0.87 |
|  | Luminal–HER2 | 0.20 (0.11 – 0.38) | <0.001 |  | 2.47 (1.17 – 5.22) | 0.019 |  | 2.64 (1.19 – 5.89) | 0.018 |
|  | HER2–Basal | 0.07 (0.01 – 0.36) | 0.002 |  | 5.34 (1.70 – 16.85) | 0.005 |  | 7.20 (2.22 – 23.35) | 0.002 |
|  | Luminal–Basal | N/A |  |  | N/A |  |  | N/A |  |
|  | Luminal–HER2–Basal | N/A |  |  | N/A |  |  | N/A |  |
|  | All non–HER2–single | 0.15 (0.09 – 0.24) | <0.001 |  | 2.04 (1.08 – 3.84) | 0.028 |  | 2.38 (1.20 – 4.70) | 0.014 |

*Abbreviations: aOR = adjusted Odds Ratio; aHR = adjusted Hazard Ratio; 95% CI = 95% Confidence Interval; p = p–value; Ref = reference; N/A = not applicable*

S**upplementary Table 3. Five–year overall and breast cancer specific survival according to BluePrint subtype**

|  |  | **5–year OS (95% CI)** | **5–year BCSS (95% CI)** |
| --- | --- | --- | --- |
| Standard subtype | HER2 | 91.8 (89.7 – 94.0) | 92.7 (90.7 – 94.8) |
|  | Basal | 80.0 (58.7 – 100.0) | 80.0 (58.7 – 100.0) |
|  | Luminal | 93.2 (88.1 – 98.6) | 94.2 (89.4 – 99.3) |
|  | All non–HER | 91.8 (86.6 – 97.4) | 92.7 (87.7 – 98.1) |
| Dual subtype | HER2–single | 92.1 (89.9 – 94.4) | 93.1 (91.0 – 95.2) |
|  | Luminal–single | 96.7 (92.2 – 100.0) | 96.7 (92.1 – 100.0) |
|  | HER2–Basal | 63.6 (40.7 – 99.5) | 63.6 (40.7 – 99.5) |
|  | All non–HER2–single | 90.7 (86.4 – 95.3) | 91.3 (87.0 – 95.7) |

*Abbreviations: OS = overall survival; BCSS = breast cancer specific survival; 95% CI = 95% Confidence Interval*

**Supplementary Table 4. Five–year overall survival according to BluePrint subtype and treatment arm**

|  |  | **No pertuzumab** | |  | **Pertuzumab** | |
| --- | --- | --- | --- | --- | --- | --- |
|  |  | **Number of events** | **5–year OS (95% CI)** |  | **Number of events** | **5–year OS (95% CI)** |
| **Standard subtype** | HER2 | 36/308 | 88.3 (84.8 – 92.0) |  | 15/313 | 95.2 (92.9 – 97.6) |
|  | Other | 5/54 | 90.7 (83.3 – 98.8) |  | 3/44 | 93.1 (86.0 – 100.0) |
| **Dual subtype** | HER2–single | 32/279 | 88.5 (84.9 – 92.3) |  | 12/278 | 95.7 (93.3 – 98.1) |
|  | Other | 9/83 | 89.2 (82.7 – 96.1) |  | 6/79 | 92.4 (86.7 – 98.4) |

*Abbreviations: OS = overall survival; 95% CI = 95% Confidence Interval*
